# Transmission of *Bartonella henselae* within *Rhipicephalus sanguineus*: Data on the Potential Vector Role of the Tick

**DOI:** 10.1371/journal.pntd.0008664

**Published:** 2020-10-01

**Authors:** Wittawat Wechtaisong, Sarah I. Bonnet, Yi-Yang Lien, Shih-Te Chuang, Yi-Lun Tsai

**Affiliations:** 1 Department of Veterinary Medicine, College of Veterinary Medicine, National Pingtung University of Science and Technology, Pingtung, Taiwan; 2 UMR BIPAR, INRAE, Ecole Vétérinaire d’Alfort, ANSES, Université Paris-Est, Maisons-Alfort, France; 3 Department of Veterinary Medicine, School of Veterinary Medicine, National Chung Hsing University, Taichung, Taiwan; University of Colorado Health Sciences Center, UNITED STATES

## Abstract

*Bartonella henselae* is a fastidious intraerythrocytic, gram-negative bacteria that causes cat scratch disease in humans. *Ixodes ricinus* has been confirmed to be a competent vector of *B*. *henselae*, and some indirect evidences from clinical cases and epidemiological studies also suggested that some other tick species, including *Rhipicephalus sanguineus*, may transmit the bacteria. *B*. *henselae* has been detected in *R*. *sanguineus* but no experimental investigations have been performed to evaluate the vector competency of this tick species regarding *B*. *henselae* transmission. To this end, this work aimed to assess the transstadial transmission of *B*. *henselae* between larvae and nymphs of *R*. *sanguineus* as well as transmission by nymphs infected at the larval stage. Four hundred *B*. *henselae* negative larvae were fed with *B*. *henselae*-infected blood by using an artificial membrane feeding system. After five days of feeding, *B*. *henselae* was detected by PCR in 57.1% (8/14) of engorged larval pools, 66.7% (4/6) of semi-engorged larval pools, and 66.7% (2/3) of larval feces pools. After molting, *B*. *henselae* DNA was also detected in 10% (1/10) of nymph pools, but not in tick feces. After a pre-fed step of nymphs infected at the larval stage on non-infected blood meal, *B*. *henselae* was detected by PCR in blood sample from the feeder, but no *Bartonella* colonies could be obtained from culture. These findings showed that *B*. *henselae* could be transstadial transmitted from *R*. *sanguineus* larvae to nymphs, and also suggest that these nymphs may retransmitted the bacteria through the saliva during their blood meal. This is the first study that validated the artificial membrane feeding system for maintaining *R*. *sanguin*eus tick colony. It shows the possibility of transstadial transmission of *B*. *henselae* from *R*. *sanguineus* larvae to nymphs.

## Author summary

*B*. *henselae* is gram-negative bacteria that infects red blood cells of humans and companion animals and causes cat scratch disease in humans. Ticks were considered to be potential vectors of *B*. *henselae* for a long time until it was finally experimentally demonstrated for *Ixodes ricinus*. Since then, no evidence on *B*. *henselae* transmission by other tick species was reported. This study was performed 1) to validate the use of artificial membrane system to feed and infect *R*. *sanguineus* ticks and 2) to determine the possibility of *B*. *henselae* transmission by *R*. *sanguineus*, a world-widely distributed ticks. Our results show that the artificial membrane feeding system can be used to maintain *R*. *sanguineus* colony in the laboratory, and that *B*. *henselae* can be acquired by *R*. *sanguineus* during a blood meal on artificial membrane feeding system and can be transmitted from larvae to nymphs that were able to inject bacterial DNA to blood during a new blood meal. However, further investigations are still needed to confirm the viability of bacteria transmitted to blood by nymphs infected at the larval stage in order to validate *B*. *henselae* transmission by *R*. *sanguineus*.

## Introduction

*Bartonella henselae* is a fastidious gram-negative bacteria which infects companion animals and is responsible for cat scratch disease (CSD) in humans [1]. Worldwide, *B*. *henselae* infection is estimated to be between 5% and 86% in cats [2–4]. *B*. *henselae* is normally transmitted from cat to cat by fleas (*Ctenocephalides felis felis*) and usually transmitted from cat to human by cat scratch due to *B*. *henselae* contamination from flea feces [5–9]. Most human CSD cases are self-limited from asymptomatic to skin inflammation, fever and lymphadenopathy [10]. However, *B*. *henselae* is becoming increasingly associated with a number of other syndromes including conjunctivitis, encephalopathy and endocarditis [11–13]. In addition, bacillary angiomatosis and peliosis hepatitis are unusual vascular proliferative lesions observed in immunocompromised patients with *B*. *henselae* infection [14, 15].

Despite cat flea has been known to be the competent vector of *B*. *henselae*, ticks have also been considered as potential vectors for *Bartonella* transmission for a long time [16–31]. *B*. *henselae* DNA has been detected in several species of ticks, including *Ixodes pacificus*, *I*. *persulcatus*, *I*. *ricinus*, and *R*. *sanguineus* [18, 26–28]. Co-infections of *B*. *henselae* with other pathogens known to be transmitted by ticks, such as *Anaplasma* spp., *Borrelia* spp. and *Rickettsia* spp., have been reported in both humans and animals by molecular evidence, suggesting a possible co-transmission of pathogenic agents after a tick bite [19, 20, 22, 25, 30, 32–34]. Moreover, *B*. *henselae* has been isolated by blood culture or detected by PCR from human patients with history of tick bites, which emphasized the hypothesis that ticks may serve as vectors for *Bartonella* spp. [16, 17, 20, 23–25, 29, 31]. In 2008, Cotté and co-workers have demonstrated that *B*. *henselae* can survive in *I*. *ricinus* during their molting from larvae to nymphs and from nymphs to adults, and can be transmitted to blood through tick saliva in an artificial membrane feeding system [35]. They also showed that the bacteria located into the salivary glands of *I*. *ricinus* adults infected at the nymphal stage was alive and infectious as when the corresponding tick salivary glands were injected to cats which then developed a bacteremia. Later, Reis and co-workers also reported that *B*. *birtlesii* could be transstadial transmitted from *I*. *ricinus* larvae to nymphs and from nymphs to adults [36]. They also demonstrated that when infected on infected mice as larvae, *I*. *ricinus* nymphs can retransmit the bacteria to naïve mice, and when infected at the nymphal stage, adults can retransmit the bacteria to blood through the membrane feeding system. All these results proved that *I*. *ricinus* can act as a vector for both *B*. *henselae* and *B*. *birtlesii* [35, 36].

The brown dog tick, *R*. *sanguineus*, has the worldwide geographic distribution. This tick plays roles as a vector of several pathogens causing clinical illness, including *Babesia vogeli* and *Ehrlichia canis* in dogs, and *Rickettsia conorii* and *R*. *rickettsii* in humans [37]. *R*. *sanguineus* has been suspected to be a potential vector of *Bartonella* spp. since 1992, when two patients developed fever with a relapsing course after tick bites, and *B*. *henselae* was isolated by culture from their blood [23]. Several molecular epidemiological surveys were then conducted, and the *B*. *henselae* DNA positive rate in *R*. *sanguineus* ticks has been reported as being 0.09% (1/209) in Italy, 3.2% (2/62) in California, USA, and 5.3% (15/281) in the central part of Taiwan [28, 30, 38]. In addition, *B*. *vinsonii* subsp. *berkhoffii* DNA has been detected in *R*. *sanguineus* adult feces suggesting that tick feces could be a potential source of *B*. *vinsonii* subsp. *berkhoffii* infection [39]. However, no validation of the vector competence of *R*. *sanguineus* for *Bartonella* spp. transmission has been performed until now.

Because of evidence of *R*. *sanguineus* harboring and potentially transmitting *Bartonella* spp. and limited information on *Bartonella* transmission by ticks, we performed the present experimental study to investigate the possibility of *B*. *henselae* transmission by *R*. *sanguineus* using an artificial membrane feeding system. This feeding technique can mimic the natural conditions of tick infection via the digestive tract in controlled condition as validated for *I*. *ricinus* with both tick-borne bacteria and parasites and, until now, was never applied to *R*. *sanguineus* feeding or infection studies [35, 36, 40–42].

## Methods

### Tick collection and population maintenance

Engorged *R*. *sanguineus* females were collected from dogs in veterinary hospitals in Taiwan and morphological identification was performed by using taxonomic keys [43]. Each engorged female was placed in a container inside a chamber with 80–90% of relative humidity, at room temperature and with a photoperiod of 16:8h (L: D) cycles until finishing her oviposition period [44]. In each larval batch, 10% of larval ticks was randomly selected and tested for *B*. *henselae* DNA presence. Only *B*. *henselae* negative offspring were then used in all experiments. After feeding and molting from the previous life stage, ticks were starved in the previously mentioned conditions for one-month until next feeding.

### *B*. *henselae* isolates

*B*. *henselae*, isolated from strayed cats in eastern part of Taiwan, was cultured on chocolate agar plates (Taiwan Prepared Media Co., LTD), at 35°C, and in an atmosphere of 5% CO_2_. Number of Colony-Forming Units (CFU) was evaluated to estimate the amount of viable *B*. *henselae*. *B*. *henselae* colonies were harvested by using sterilized polypropylene loop (SPL Lifesciences Co., LTD), suspended in sterile 1x of Phosphate-Buffered Saline (1x PBS), and used immediately for tick feeding [35]. DNA of *B*. *henselae* used as a positive control for nested-PCR was extracted from a bacterial colony using DNeasy Blood & Tissue kit (Qiagen).

### Skin membrane preparation

Outbred ICR (Bltw:CD1) mice (BioLASCO Taiwan Co., LTD), around 10 weeks old, without any treatment from cooperating laboratories in National Pingtung University of Science and Technology, Taiwan were used to obtain skins. Mice skins with the dermal thickness around 300 μm were considered to use in artificial feeding of *R*. *sanguineus* larvae and nymphs (hypostome length: 50 μm and 120 μm, respectively) [45, 46]. The mice skins were then processed as previously described [41]. Briefly, dissected skins were first sterilized in 70% ethanol for 5 min, followed by rinsing in sterile distilled water for 5 min and then 1x PBS for 5 min. Those skins were finally aseptically treated in an antimicrobial solution of Gentamicin (10 mg/ml), Amphotericin B (0.25 μg/ml), Penicillin (50 U/ml) and Streptomycin (50 μl/ml) for 10 min. All dissected skins were then stored at -20°C not longer than 3 months until use.

### Blood preparation for tick feeding

Goat blood, obtained from a goat farm in Pingtung, Taiwan and confirmed *Bartonella* DNA negative by PCR, was used in all following experiments (ethical permit IACUC number: NPUST-105-036). After collection, the blood was defibrinated and depleted of functional complement by heat treatment at 56°C for 30 min [35]. To prevent fungal and bacterial contamination during tick feeding, decomplemented blood was supplemented with 20 μl/ml of Fosfomycin, 0.25 μg/ml of Amphotericin B, and 10 KU/ml of Heparin [35].

### Feeding larvae with *B*. *henselae*-infected blood

**Fig 1:**
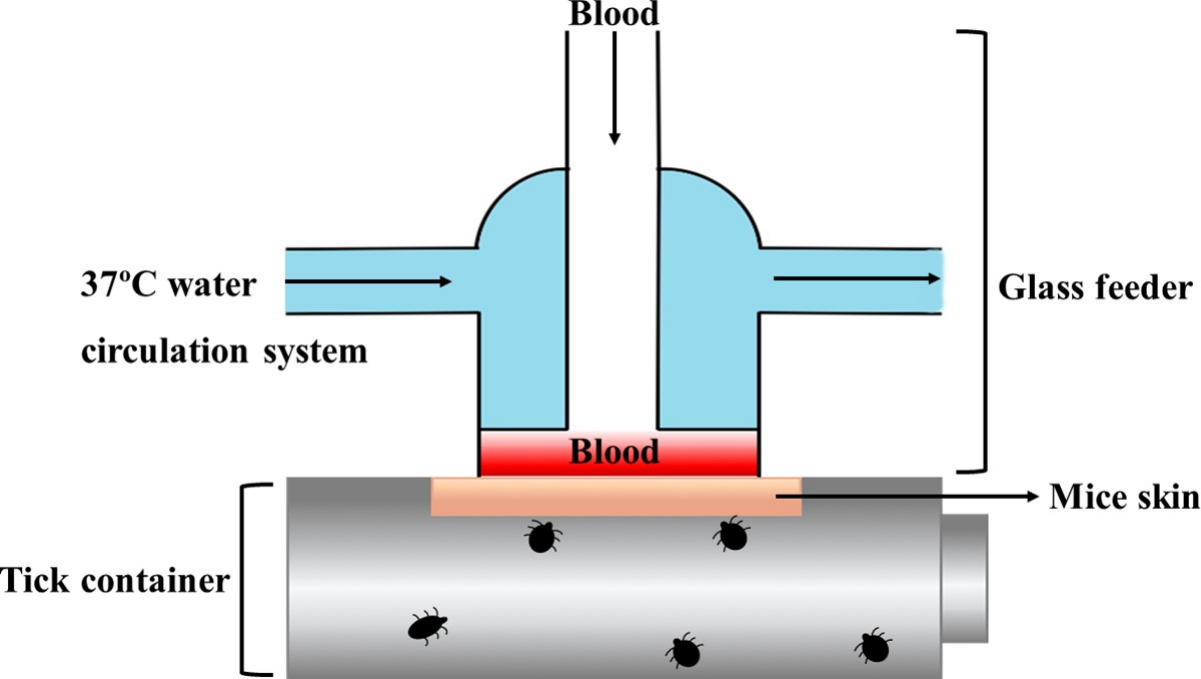
Diagram of an artificial membrane feeding system used to feed *R*. *sanguineus* larvae and nymphs. The artificial feeder consists of a glass feeder, animal skin, and tick container. The glass feeder is connected to a 37°C water circulation system to mimic host body temperature.

The artificial membrane feeding system was adapted from Bonnet *et al* [41]. This system consists of a glass feeder closed with a mice skin at the bottom, and placed on the top of the tick container. Placing the blood above the skin membrane supports a continuous gravitational pressure on the membrane. The glass feeders were connected to a 37°C water circulation system to mimic host body temperature, attract ticks and preserve *Bartonella* in the blood (Fig 1). This system was established under room temperature, 80–90% of relative humidity and photoperiod of 16:8h (L: D).

**Fig 2:**
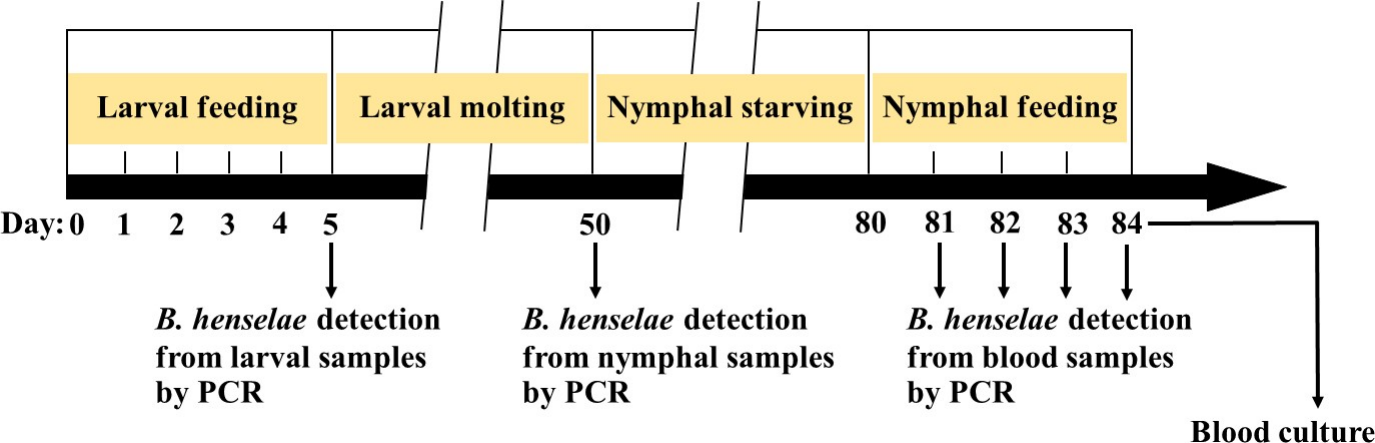
Duration of tick feeding experiment of *R*. *sanguineus* larvae into nymphs. Larvae were fed for 5 days and were allowed to complete their molting period, which ranged from 15–45 days. After they become nymphs, they were starved around one month and then fed for 4 days. *B*. *henselae* were detected by PCR assay from larval samples (at the end of larval feeding), nymphal samples (after molting completed) and blood samples (daily collected during the nymphal feeding). After 4 days of nymphal feeding, blood from each feeder was collected for *Bartonella* isolation.

The general framework of the tick feeding experiment is shown in Fig 2. A total of 400 larvae were fed with *B*. *henselae*-infected blood for 5 days by using artificial membrane feeding system under laboratory conditions as described above. The larval feeding duration in the present study was adjusted from the reported time of feeding of *R*. *sanguineus* larvae on rabbits under laboratory conditions, i.e. 3 to 6 days [44]. The *B*. *henselae*-infected blood was prepared as follow: a total of 40 μl of *B*. *henselae* suspension at a concentration of 10^9^ CFU/ml was added to 4 ml of decomplemented blood for feeding. Blood was changed twice a day at 12 h intervals, and the side of mice skin in contact with the blood was washed three times with RPMI 1640 (CORNING) [41]. As a control group, 200 larvae were fed on non-infected blood under the same condition and the blood was changed once a day (Fig 2). At the end of feeding experiment, larvae that spontaneously detached from mice skin were considered as engorged larvae, and the larvae that were still attached on the mice skin and then manually detached were considered as semi-engorged larvae. The attachment rate of *R*. *sanguineus* larvae on mice skin was calculated at day 5 after the beginning of the experiment.

### Feeding nymphs with non-infected blood

A total of 60 nymphs that molted from experimentally *Bartonella*-infected larvae were fed in a unique feeder with non-infected blood for 4 days according to the delay of bacteria detection in blood samples reported for *I*. *ricinus* [35, 36]. As control, 40 nymphs molted from larvae engorged on non-infected blood were also fed in another feeder with non-infected blood. A total of 100 μl of blood was collected daily from each feeder and used to detect *Bartonella* DNA by PCR. After 4 days of feeding, 10 μl of blood was spread on chocolate blood agar plates to detect *Bartonella* colonies [35]. At day 4, nymphs were manually detached on the mice skin and considered as semi-engorged nymphs for calculating the nymph attachment rate (Fig 2).

### DNA extraction from tick and blood samples

DNA was extracted using DNeasy Blood & Tissue kit (Qiagen) from 6 kinds of samples, including 100 μl of blood samples, pools of 5 engorged larvae, pools of 10 semi-engorged larvae, pools of larval feces, pools of 3 unfed nymphs (molted from engorged larvae) and pools of nymphal feces from both experimental and control groups. Tick feces were collected from the tick container during the feeding process by sterilized polypropylene loop and needle (SPL Lifesciences Co., LTD), and suspended in 100 μl of sterile 1x PBS. The final elution was 50 μl for all samples. All DNA samples were stored at -20°C until PCR processing for *B*. *henselae* DNA detection.

### *B*. *henselae* PCR amplification

*B*. *henselae* DNA was detected by using nested-PCR [47]. The primers forward 5’-CTTCGTTTCTCTTTCTTCA-3’ and reverse 5’-CTTCTCTTCACAATTTCAAT-3’ used for outer reaction, amplified fragments of the 16S-23S rRNA internal transcribed spacer (ITS) region of *Bartonella* spp. [48]. The primers forward 5’-TTGCTTCTAAAAAGCTTATCAA-3’ and reverse 5’-CAAAAGAGGGATTACAAAATC-3’ used for inner reaction were designed to be specific to *B*. *henselae* amplifying a 254-bp fragment [47]. PCR mixtures was set up as follows: 5 μl of DNA template, 1 μl of 10 μM of each primer, 5 μl of 10X *Taq* buffer (Genomics BioSci & Tech, Taiwan), 4 μl of 2.5 mM of dNTPs Mixture (Genomics BioSci & Tech, Taiwan), 1 μl of 2.5 U/μl of *Taq* DNA polymerase (Genomics BioSci & Tech, Taiwan), and adjusted to a final volume of 50 μl with distilled water. The PCR conditions for both outer and inner reactions were those described by Sato and co-workers [47].

### Sequence and statistical analyses

The *B*. *henselae* suspected positive samples were sent for nucleotide sequencing (Genomics BioScience and Technology Co., Ltd., Taiwan). Sequence data was analyzed for genetic relationship in GenBank database by using NCBI nucleotide BLAST tool and validated sequences were aligned and analyzed by using MegAlign (DNASTAR, Inc., WI, USA). The attachment rates of *R*. *sanguineus* larvae and nymphs, and the engorgement rates of *R*. *sanguineus* larvae in control and experimental groups were calculated, and compared by using the Fisher’s exact test (p<0.05) (GraphPad Prism 8.4.2 software). *B*. *henselae* infection rates in tick pools were evaluated by using the bias-corrected maximum likelihood estimation (MLE) method with 95% confidence interval, analyzed per 100 ticks by the PooledInfRate statistical software version 4.0 [49].

## Results

### High attachment rates of *R*. *sanguineus* on artificial membrane feeding system

**Fig 3:**
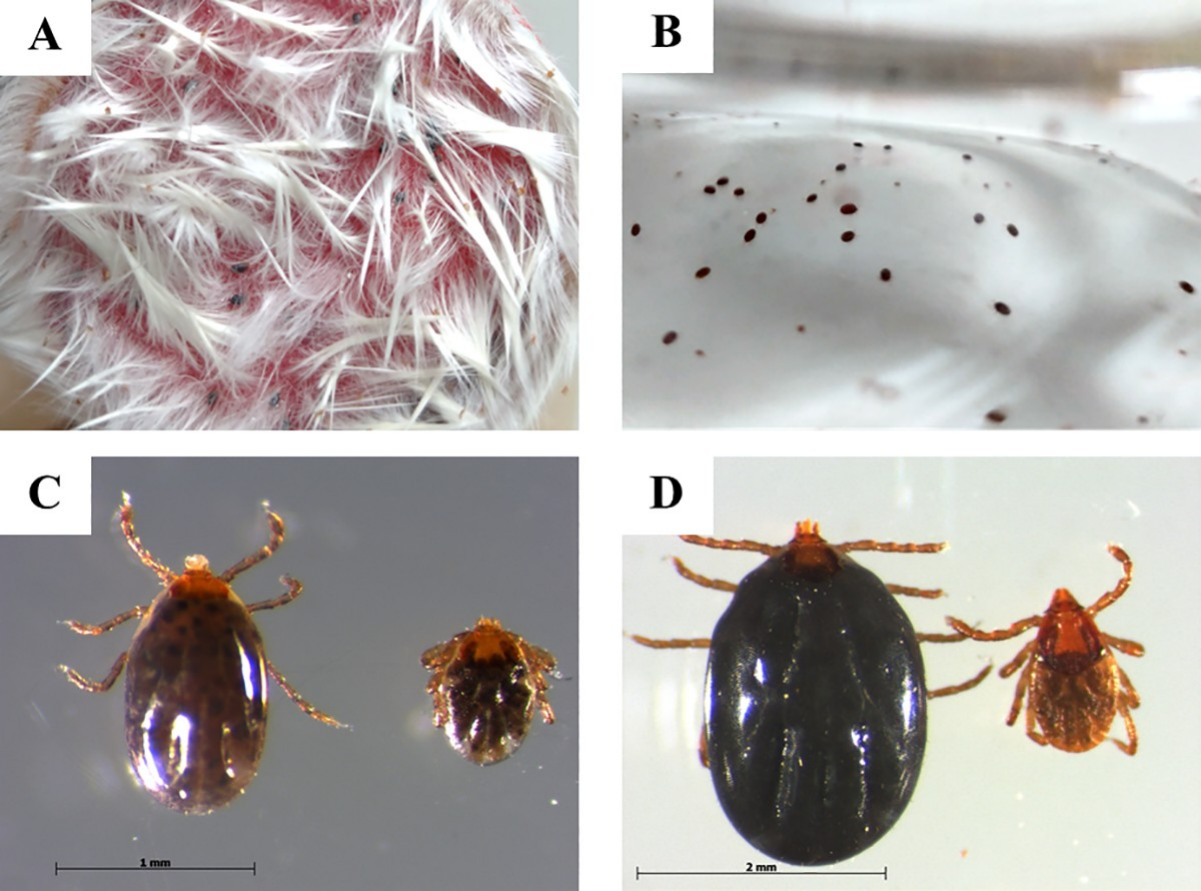
*R*. *sanguineus* larvae and nymphs engorged using goat blood infected or not with *B*. *henselae*. (A) Larvae attached on the mice skin. (B) Engorged larvae detached from the mice skin after 3 days. (C) Engorged larva (left) and semi-engorged larva (right). (D) Semi-engorged nymph (left) and unfed nymph (right).

In the present study, some of *R*. *sanguineus* larvae started to attach on mice skin around 6 hours after tick placement, and some engorged larvae from both groups started to detach spontaneously on day 3 (Fig 3A and 3B). After 5 days of feeding on *B*. *henselae*-infected blood, a total of 43% (172/400) of larvae were completely engorged, and 41.5% (166/400) were semi-engorged (Table 1 and Fig 3C). For control group, 34% (68/200) and 47.5% (95/200) of larvae were fully engorged and semi-engorged, respectively. The engorgement rate of larvae from experimental group (43%) was significantly higher than in control group (34%) (p = 0.0344). Attachment rates of *R*. *sanguineus* larvae on mice skins in experimental and control groups were 84.5% and 81.5%, respectively, with no significant difference (p = 0.3529) (Table 1). Engorged larvae were maintained under rearing conditions until they completed their molting period, which ranged from 15–45 days. Regarding nymphs on day 4 of feeding, 56.7% (34/60) of nymphs from experimental group and 70% (28/40) of nymphs from control group were attached on mice skins and considered as semi-engorged nymphs (Table 1 and Fig 3D). Attachment rates were not significantly different between nymphs from experimental group and that from control group (p = 0.2908).

**Table 1:** Attachment rates of *R*. *sanguineus* larvae and nymphs fed on mice skin by artificial membrane feeding system.

Ticks fed on mice skin	*Larval stage*		*Nymphal stage*	
	Experimental group (400)	Control group (200)	Experimental group (60)	Control group (40)
**No. of engorged ticks (%)**	172 (43%) *	68 (34%) *	-	-
**No. of semi-engorged ticks (%)**	166 (41.5%)	95 (47.5%)	34 (56.7%)	28 (70%)
**Others (%)**	62 (15.5%)	37 (18.5%)	26 (43.3%)	12 (30%)
**Attachment rate**	338 (84.5%)	163 (81.5%)	34 (56.7%)	28 (70%)

Data were analyzed statistically to compare results between ticks fed on *B*. *henselae*-infected blood (experimental group) and non-infected blood (control group) by Fisher’s exact test (* p<0.05).

Others: unfed, inactive, or died ticks.

### *B*. *henselae* DNA detection in engorged *R*. *sanguineus* larvae and transstadial transmission to nymphs

**Fig 4:**
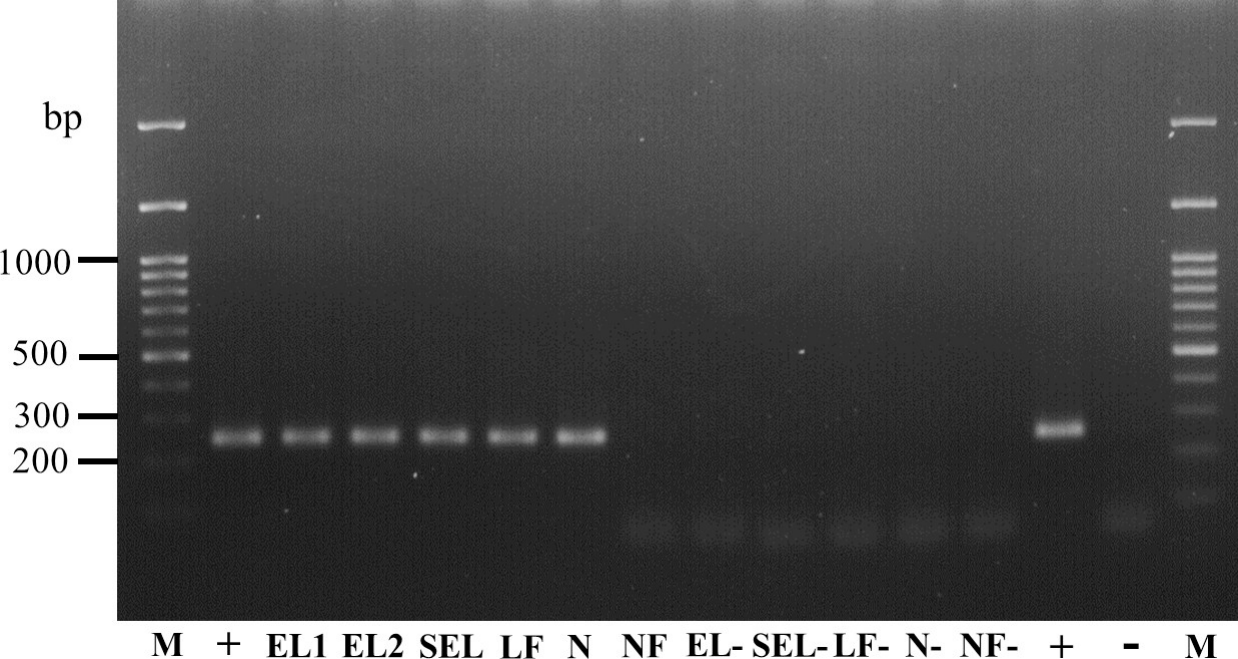
*B*. *henselae* DNA detection in *R*. *sanguineus* larvae and nymphs. Detection of *B*. *henselae* DNA by nested-PCR in representative samples: M, DNA marker; EL1 and EL2, pooled engorged larvae (Experimental group); SEL, pooled semi-engorged larvae (Experimental group); LF, pooled larval feces (Experimental group); N, pooled unfed nymphs (Experimental group); NF, pooled nymphal feces (Experimental group); EL-, pooled engorged larvae (Control group); SEL-, pooled semi-engorged larvae (Control group); LF-, pooled larval feces (Control group); N-, pooled unfed nymphs (Control group); NF-, pooled nymphal feces (Control group); +, positive control (*B*. *henselae* DNA); -, negative control (distilled water).

Pools of engorged larvae (N = 5), pools of semi-engorged larvae (N = 10), and pools of larval feces were collected and tested for *B*. *henselae* DNA presence after 5 days of feeding on infected blood. PCR result showed that 57.1% (8/14) of engorged larval pools presented the expected *B*. *henselae*-specific 254-bp DNA fragment. 66.7% (4/6) of semi-engorged larval pools and 66.7% (2/3) of larval feces pools from the experimental group were also positive for *B*. *henselae* DNA. The MLE of *B*. *henselae* infection rate among pools of engorged and semi-engorged larvae were then estimated as 14.94% (95% CI, 7.30–27.59%) and 9.06% (95% CI, 3.15–23.79%), respectively. Samples from the control group were all negative for *B*. *henselae* detection (Table 2 and Fig 4).

**Table 2:** Detection of *B*. *henselae* DNA in *R*. *sanguineus* larvae engorged on infected blood and in nymphs after molting and maximum likelihood estimation (MLE) of tick infection.

Pooled samples	No. of individuals	No. of positive pools/no. of pools tested (%)	Maximum likelihood estimation (MLE)	95% CI
***Experimental group***				
Engorged larvae	70	8/14 (57.1)	14.94%	7.30–27.59%
Semi-engorged larvae	60	4/6 (66.7)	9.06%	3.15–23.79%
Larval feces		2/3 (66.7)	-	-
Unfed nymphs	30	1/10 (10)	3.33%	0.20–15.30%
Nymphal feces		0/3 (0)	-	-
***Control group***				
Engorged larvae	10	0/2 (0)		
Semi-engorged larvae	20	0/2 (0)		
Larval feces		0/2 (0)		
Unfed nymphs	12	0/4 (0)		
Nymphal feces		0/2 (0)		

95% CI: 95% confidence interval.

After larvae molted to nymphs, some of the unfed nymphs and their feces were tested for *B*. *henselae* DNA detection by PCR. One (10%) pooled nymph sample was detected positive for *B*. *henselae* DNA, when pooled nymphal feces samples from the experimental group were all negative. The MLE of *B*. *henselae* infection rate of pooled nymphs was then evaluated as 3.33% (95% CI, 0.20–15.30%). *B*. *henselae* DNA was not detected in samples from the control group (Table 2 and Fig 4). All obtained sequences showed 100% identity to each other as well as with the partial sequence of 16S-23S ribosomal RNA intergenic spacer *B*. *henselae* (Accession number MN170540.1).

### *Bartonella* DNA detection in blood during feeding of nymphs infected at the larval stage

**Fig 5:**
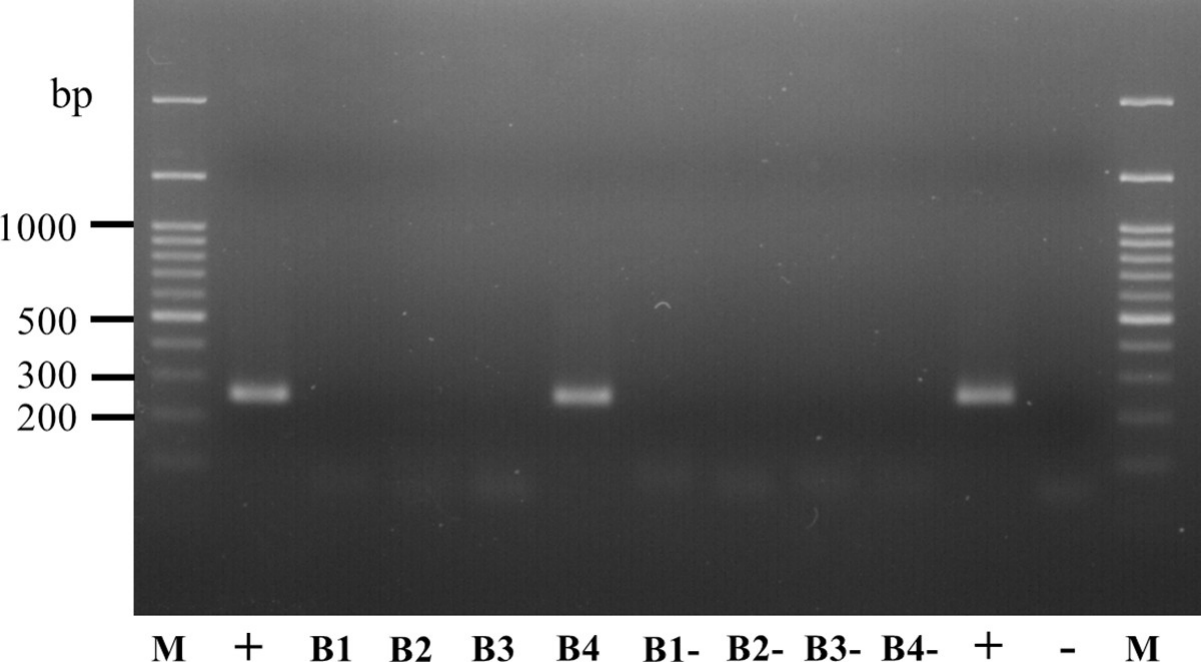
Detection by nested-PCR of *B*. *henselae* DNA in non-infected blood after feeding of nymphs infected at the larval stage. M, DNA marker; B1—B4, blood samples collected from day 1 to 4, respectively (Experimental group); B1- - B4-, blood samples collected from day 1 to 4, respectively (Control group); +, positive control (*B*. *henselae* DNA); -, negative control (distilled water).

Sixty nymphs from the experimental group infected as larvae and forty nymphs from the control group were fed separately on non-infected blood for 4 days. To determine whether *B*. *henselae* was transmitted by nymphs through blood sucking, blood samples were collected daily and tested for *B*. *henselae* DNA. The PCR result showed that *B*. *henselae* DNA was detected in blood 4 days after nymph attachment to the skin for those molted from larvae engorged on infected blood, when PCR were all negative for the control group (Fig 5). Unfortunately, no colonies were obtained after 14 days of culture of the blood sample taken on day 4, as for control.

## Discussion

In the present study, an artificial membrane feeding system using mice skin and initially designed for *I*. *ricinus* was successfully set up for *R*. *sanguineus* [41]. Our results show high larval attachment rate of 81.5% in the control group and 84.5% in the experimental group, as well as 70% in the control group and 56.7% in the experimental group for nymphs. Under similar feeding conditions and treatments, the engorgement rate of *R*. *sanguineus* larvae (43%; 172/400) in experimental group shown in the present study were lower than those (81% and 84.5%) reported in previous studies for *I*. *ricinus* [35, 41]. For this last tick species, it has been demonstrated that blood origin (chicken or sheep) does not impact the proportion of engorged ticks, the weight of engorged ticks or the duration of feeding [50]. It is therefore conceivable that, despite we used different blood origin, the difference observed here is due to the tick species, including for example differences in appetite for mice skin, or into the length of tick mouthparts, which are 50 μm and 90 μm in the larvae of *R*. *sanguineus* and *I*. *ricinus*, respectively [45].

*B*. *henselae* DNA was detected in pooled engorged and semi-engorged larvae fed on *B*. *henselae*-infected blood, which supports the hypothesis that *Rhipicephalus* ticks could acquire *Bartonella* or at least *Bartonella* DNA during their feeding, as already suggested in some epidemiological studies [28, 30, 38]. In this study, the engorgement performance of larvae fed on *B*. *henselae*-infected blood (43%) was higher than on non-infected blood (34%). This finding has also been found for some other vector-borne pathogens, such as *Plasmodium* spp. and *Trypanosoma* spp., suggesting that the infectious status of the hosts can enhance feeding behavior of their vectors [51–54]. However, to the contrary, it has been reported that for *I*. *ricinus*, feeding on a *B*. *henselae*-infected blood meal through membrane feeding or on infected mice decreased the proportion and the weight of engorged ticks, but did not affect tick feeding duration [50]. The same trend is found here for the nymphs with a higher attachment rate for the control group, which might suggest that tick infection perturbs their feeding. However, experiments involving a higher number of ticks with sufficient time of feeding for their completely engorgements would be necessary to confirm this hypothesis. In addition, obtaining the weight of ticks by measuring the amount of ingested hemoglobin using spectrophotometry in future studies can provide a quantitative evaluation of the ticks’ fully engorged and semi-engorged status and the pathogen uptake by ticks [55, 56].

The presence of *B*. *henselae* DNA in nymphs molted from larvae engorged on infected blood suggests that *B*. *henselae* could be transstadially transmitted from larvae to nymphs during the molting process. This possibility is supported by our results showing that nymphs infected at the larval stage are able to inject the bacterial DNA into blood during their blood meal. Indeed, such a result suggests that DNA is injected through the saliva, and that the bacteria is then present into the salivary glands of the tick infected as the previous life stage, as demonstrated for both of *B*. *henselae* and *B*. *birtlesii* in *I*. *ricinus* ticks [35, 36]. It is therefore more than likely that it is viable bacteria, which escaped from the tick digestive tract and then invaded the salivary glands of nymphs. Transmitted pathogens need to effectively to hide or defeat on defense mechanisms in tick midgut, which is the first tick-pathogen interaction [57, 58]. The pathogens eventually migrate to tick ovaries for promoting transovarial transmission, or migrate to salivary glands for being transmitted to a host through tick saliva [57, 58]. Thus, although the bacteria present in the blood from the feeders could not be cultivated, our results provide strong assumptions about the ability of *R*. *sanguineus* to transmit *B*. *henselae* during the bite.

One possible reason for failure to obtain bacterial colonies can be that the number of viable bacteria from the collected sample was too low as only 10 μl out of the 4 ml of blood from each feeder was used for culture. Once ticks are feeding on an artificial membrane, a few microliters of tick saliva are mixed with the blood in the feeder, which possibly reduce the bacterial concentration below the detectable level [36]. In the study of Cotté and co-workers, some *B*. *henselae* colonies were obtained from 10 μl of blood taken from the feeder 84 hours after *I*. *ricinus* attachment, but that study concerned females infected at the nymphal stage, which inject more saliva during their feeding than nymphs and then possibly more bacteria [35]. To validate or not the viability of the bacteria presents into the blood of the feeder after *R*. *sanguineus* nymphal feeding, a higher volume of blood should be then tested in culture. Another hypothesis is that the bacteria, although alive to join the salivary glands, was however destroyed by the tick immune system in the salivary glands, as for example, by the salivary 5.3kDa antimicrobial protein (ISAMP), which has the ability to kill gram-positive and gram-negative bacteria [59]. In this case, this explanation echoed the hypothesis of selective adaptation of *Bartonella* spp. In fact, several arthropod vectors may harbor a wide range of *Bartonella* spp. but may not be efficient vectors for transferring all these *Bartonella* spp. to their hosts [60].

The presence of *B*. *henselae* DNA in larval feces samples also suggest that *B*. *henselae*-contaminated tick feces could be a potential source of *Bartonella* infection for humans or animals as it is the case for fleas [5–9]. However, no *B*. *henselae* DNA was detected in nymphal feces suggesting that bacteria may not stay in tick digestive system during the tick molting period. However, further investigations, confirming the viability of *B*. *henselae* from tick feces by culture, are required to understand the role of tick feces in *Bartonella* transmission. In a previous study, *B*. *vinsonii* subsp. *berkhoffii* DNA has been detected in *R*. *sanguineus* tick feces after post-capillary tube feeding, but no viable *Bartonella* could be isolated because of bacterial and fungal contamination [39]. Even if *in vivo* experiment has also showed that no dogs became bacteremia nor seroconverted when inoculated with tick feces collected from *Bartonella*-fed ticks, tick feces could not be ruled out as a potential source of *Bartonella* infection [39].

In conclusion, our findings demonstrated that the membrane feeding system could be successfully used to engorge and maintain *R*. *sanguineus* colony in the laboratory. Such a development now opens up real perspectives in the study of *R*. *sanguineus*, its biology, its vector competence, as well as in the study of molecular interactions with the transmitted pathogens. Our results also support that *B*. *henselae* could be transstadial transmitted from *R*. *sanguineus* larvae to nymphs. However, future both *in vitro* and *in vivo* studies would need to be performed to clarify more evidence of the vector competence of *R*. *sanguineus* larvae, nymphs, and adults for *B*. *henselae*. Despite having probably low epidemiological importance, the demonstration of such competence would first emphasize, another time, broad arthropod host range for bartonellae. Secondly, it would explain some cases of bartonellosis in patients, especially to include the diagnosis of bartonellosis following *R*. *sanguineus* tick bites, which is the most widespread tick in the world.
